# How *Bdellovibrio bacteriovorus* became a model for invasive bacterial predation

**DOI:** 10.1128/jb.00137-26

**Published:** 2026-09-18

**Authors:** Andrew L. Lovering, R. Elizabeth Sockett

**Affiliations:** 1 School of Biosciences, University of Birmingham 85442, Birmingham, United Kingdom; 2 School of Life Sciences, University of Nottingham 105592, Nottingham, United Kingdom; Dartmouth College Geisel School of Medicine, Hanover, New Hampshire, USA

**Keywords:** *B. bacteriovorus*, predatory lifestyle, history

## Abstract

*Bdellovibrio bacteriovorus* is a gram-negative Proteobacterium that has evolved to use a novel, predatory, intrabacterial lifestyle. Once encountered under a microscope, in a lecture, or a textbook, it is seldom forgotten. *B. bacteriovorus* speedily collides with, enters, modifies, and kills other gram-negative bacteria. Residing inside the periplasm of the dead prey for several hours, it uses prey molecules for growth and replication, only bursting out from that temporary home when its progeny are fully formed predators themselves. Rapid motility whilst hunting contrasts with sessility during prey consumption—these key behaviors, studied by early researchers, have been followed by molecular studies in recent times. Genomic analysis has enabled the linkage and discovery of proteins involved in key regulatory circuits, allied to enzymology describing prey cell manipulation and deconstruction. Indeed, the prey bacterium of a *B. bacteriovorus* is the natural “petri dish” of suitable components for predator growth. A fascinating *B. bacteriovorus* conundrum continues to be: “how does a predator consume materials very similar to those from which it is built, without self-damage?”. More has been discovered and is yet to be learned about these bacterial damaging enzymes: how predators use them in nature and whether they can be applied to assist human conflicts with pathogens. However, the elegance and molecular complexity, with which *B. bacteriovorus* accomplishes its intrabacterial lifestyle, will continue to challenge researchers and present new biology in the 21st century. New discoveries, tools, and approaches position *B. bacteriovorus* research for many more breakthroughs.

## INTRODUCTION

*Bdellovibrio bacteriovorus* is an agile, comma-shaped, uniflagellate bacterium which invades, chemically modifies, kills, and internally consumes other gram-negative bacteria, bigger than itself. Whereas *Escherichia coli* has been described, in the first mini-review of this series, as the “flagship bacterium of molecular biology,” the predatory *B. bacteriovorus* represents a fascinating “pirate ship” of the bacterial fleet—invading, colonizing, and looting from its temporary home in the periplasm of prey bacteria. The biochemical and geographical complexity of its lifestyle, consuming materials similar to its own, without damage, and managing entry to, and exit from, another bacterium has fascinated microbiologists for over 60 years. It also continues to provide “new biology” from which previously uncharacterized antibacterial or biotechnological processes can be discovered. The application of *Bdellovibrio* cells against pathogenic infections is one of the major motivations for predator studies, and such research warrants a whole review of its own. Here, we attempt to highlight some historic discoveries in *Bdellovibrio* and where that research has brought our understanding of predatory *B. bacteriovorus* biology today.

From a soil sample in Berlin, *B. bacteriovorus* entered the study of microbiology, forming clear areas on otherwise opaque petri dish lawns of *Pseudomonas phaseolicola* bacteria grown by Heinz Stolp in an intended bacteriophage study in 1962 (1, 2). The clear plaques formed more slowly than those expected by bacteriophage infection, taking several days to appear and continuing to enlarge. Purifying, microscopically examining, and culturing their contents revealed not bacteriophage but instead fast-swimming, tiny 0.3 × 1.0 μm, comma-shaped gram-negative bacteria, invading and killing the *P. phaseolicola*. Stolp subsequently established that *B. bacteriovorus* had a broad, invasive prey range across different gram-negative bacteria (2).

Appropriately named via both Greek and Latin, and 1800s microbial terminology, as *Bdellovibrio* “quivering curved leech,” *bacteriovorus* “bacteria-eater,” it entered microbiological consciousness with myriad scientific questions. These included the following:

How are prey bacteria found and entered?

How soon do prey die?

What are the predatory enzymes?

How can a prey cell be modified for occupation by a predator?

How does a predatory bacterium safely interact with and digest a prey cell?

What controls transitions between hunting and killing prey versus living alongside them?

*B. bacteriovorus* strains 109J and HD100 have been the most researched strains; derived from soil and sewage during the early studies of Stolp and Starr (3). Strain 109J was the most studied in the early years. Oral stories report it was culturally passaged biweekly on laboratory prey such as *E. coli* for many years, as was common lab practice (pers comm). Strain HD100 was less often cultured and studied initially but maintained as a frozen culture in the DSMZ German culture collection. It was chosen from there for genome sequencing (facilitated using *B. bacteriovorus* cultured on a *Pseudomonas* prey bacterium with different DNA GC content) (4).

Ribosomal RNA gene and genomic sequencing of both strains (4–6) allowed classification initially of *B. bacteriovorus* as a delta proteobacterium (5) with some gene similarities to Myxobacteria, but then more recently, it has been placed into a more distinct phylum, the Bdellovibrionota (7). Both 109J and HD100 strains are similar genetically (8) and physiologically, with HD100 having the comma-shaped cell and a faster flagellar motility and invasion speed (9), but both carrying out the same invasion process on a range of gram-negative bacteria.

Many talented pioneers joined the early days of *Bdellovibrio* research in the 1960s, often using *E. coli* as a laboratory prey—*E. coli* remains a common choice for prey in many modern studies, although one should reflect on how often these two are likely to interact in the environment. As a soil and aquatic bacterium, *B. bacteriovorus* has evolved alongside many different environmental prey, as the works of Henry Williams and colleagues and Laura Williams and colleagues have shown (10–12). After discovering *B. bacteriovorus*, Stolp moved to California and worked with Mortimer Starr at UC Davis, establishing that diverse gram-negative bacteria could be killed and that *B. bacteriovorus* could be isolated from natural environments, including diverse soil and water samples (3). His foundational and pioneering work led to the award of the prestigious Robert Koch Prize to Stolp in 1968, after his return to Germany, and to the first microscopic, energetic, and ecological understandings of *B. bacteriovorus* and the development of predatory bacterial studies (2, 13, 14). Starr and his colleagues Baigent, Seidler, and Snellen then went on to determine the first invasive details of *B. bacteriovorus* predation, details of its flagellum, some of its bacteriolytic enzymes, and its contacts at the prey surface, naming the predator-containing, dead prey cell a “bdelloplast” (15–20).

Longstanding *B. bacteriovorus* pioneer Edouard Jurkevitch, whose research is discussed later, recounted details that he gained firsthand from Heinz Stolp and other pioneers, of the discovery era (21). He describes how Moshe Shilo met *B. bacteriovorus* in Starr’s lab, on a visit to UC Davis. Shilo credited his sabbatical host Roger Stanier for suggesting *Bdellovibrio* to him as a research subject (22). Shilo started, with Mazal Varon, extensive work on the cell biology, kinetics, and ecology of predation and of prey-independent culturing, vital to later genetic and transcriptional studies of predatory mutants (22–30). American Sydney Rittenberg met *B. bacteriovorus* in Shilo’s lab, in Israel despite being from California (21). They published a paper together, with Varon (31), measuring parameters of predator-caused damage to prey membranes and bioenergetic capabilities, something that is still difficult to assess today. Rittenberg and his graduate students and researchers, including Michael and Linda Thomashow, Robert Hespell, and Ned Ruby (21), made great strides in understanding the physiology and biochemistry of *B. bacteriovorus* predation. Thanks to them, we began to understand the staged predatory cell cycle (31, 32), some of the enzymes of predation (31, 33–38), details of their flagellum (39–41), their predatory metabolism (42–45), energetics, prey wall modifications, and signaling (46, 47). Mortimer Starr became busy with plant pathogen work, and after initial important discoveries, other American and Israeli labs, including later that of Edouard Jurkevitch and newer colleagues in Asia, Canada, Europe, and the United States, took up the torch of *B. bacteriovorus* studies (21).

It is humbling to read their papers and those by Abram and Davis, and Samuel Conti’s lab (48–51) including Conti’s colleagues Diedrich and Tudor. They began working when bacterial physiology , cell fractionation, microscopy, and protein analysis were advancing across bacteriology. Thanks to them, we began to see the complex invasion process (48, 49), how that can be paused in some encysted species (50), and some of the challenges of discerning predator from prey components (51). The early researchers did so much, in such a complex bacterium, without the benefit of genomes and post-genomics, almost fishing in the dark to discover biological function. By 1973 Heinz Stolp (2) was able to review the developing field that he had begun. It was their inspiring papers that made several labs take up *Bdellovibrio* research at the turn of the millennium, when methods and background data were certainly more helpful. The sequencing of the first *B. bacteriovorus* genome by Rendulic, Jagtap, Schuster, and colleagues provided a real breakthrough, detailing a large 3.78 Mb genome encoding approximately 3,584 genes, including many predatory enzymes, and allowing forward genetics to test predatory function (4). Subsequent genomic comparisons and further genome analysis of *B. bacteriovorus* (6, 8, 11, 52) showed similarities across predatory bdellovibrios. Genomics aided their designation, initially as Deltaproteobacteria, then as members of the class Oligoflexia (53) within the Proteobacteria, with the International Committee on Systematics of Prokaryotes, then reclassifying them into phylum *Bdellovibrionota* (7).

Studying the interaction of *B. bacteriovorus* and prey microscopically, a distinctive feature is the very rapid swimming motility and then collision and temporary stationary attachments to prey by *B. bacteriovorus* (1, 3). Excellent electron microscopy in the 1960s/1970s showed that *B. bacteriovorus* have a single membrane-sheathed flagellum (15, 49) and an unusual tapering helical flagellar filament wave shape within it (41). The filament was later found, by Lambert, Aizawa, and colleagues, to consist of six different flagellin proteins (54, 55). Prey interactions were always at the non-flagellate predator pole, and initial proposals came about that flagellar rotation could push predators to drill into prey for entry (49). Later, this was shown not to be the case, with flagellar filament-minus *B. bacteriovorus* still entering prey if they were physically pipetted onto them (55), hence suggesting that flagellar propulsion was for prey location only. Research by Camilli, Kadouri, Shanks, and colleagues showed that significant motility of the prey itself could negatively inhibit predator attachment (56).

While it is still not clear what chemotactic stimuli might bring *B. bacteriovorus* towards prey, Conti’s lab showed that it could bias flagellar swimming to respond to a range of amino acids and organic acids (57–60). Lambert and colleagues (61) showed that chemotaxis by *B. bacteriovorus* played a role in the efficient location of environments rich in prey bacteria. The sensory thresholds and kinetic data of bacterial chemotactic signaling, analyzed and interpreted in other bacteria by physicist Howard Berg (62–64), aid this conclusion. Berg explained that given the swimming and sensing speeds of bacteria, it is not possible for chemotactic sensing of prey to occur sensitively and quickly enough to govern a direct encounter. This applies to encounters between a *B. bacteriovorus* cell and a single prey cell. This is because chemotaxis leads to overshooting and circling back around a stimulus, rather than going right to it. So, *B. bacteriovorus*, with its fast swimming speed of 60–100 µm/sec (55, 65, 66), can use chemotaxis for the more distant location of prey-rich areas, within which they swim quickly, making random collisions with prey, at close quarters, that are then more frequent and so more productive. The *B. bacteriovorus* HD100 genome has tens of putative chemoreceptors, underscoring that chemotaxis has some importance in predatory life. It will be interesting to see if *B. bacteriovorus* uses any in a repellent sensing fashion to avoid areas of likely non-prey or already-invaded prey.

After collision with prey, the predator flagellum stops rotating, and temporary external attachment to prey occurs. Abram and Burnham noted thin *Bdellovibrio* filaments contacting the prey surface (48, 49). Later, Evans and colleagues and Mahmoud, Koval, and colleagues discovered that Type IV pili are required, likely participating in the attachment and possibly active inward movement at prey invasion (67, 68). Retractable pilus fibers are known to allow locomotion of bacteria on surfaces and in biofilms (69). Without the main *B. bacteriovorus* pilus fiber gene *pilA*, invasion and predatory growth did not occur, although attachment to prey was still observed (68); antibodies against pilus proteins were demonstrated to prevent prey invasion (67).

Another mode of movement, gliding motility on solid surfaces, is also exhibited by *B. bacteriovorus* and likely aids predation on biofilms, which was documented by Kadouri and O’Toole (70). Work by Lambert, Lai, Huwiler and colleagues showed that five copies of gliding apparatus operons are expressed at different times during the predatory cycle (71, 72, 73). Their differential expression suggests that gliding motility is important during different aspects of the predatory lifecycle. Lambert, Milner, and colleagues show in supplementary movies as part of their publications that gliding on surface cultures can allow prey invasion and prey exit without flagellar motility and that gliding requires cell polarity signaling including by the MglA GTP-binding protein, which is better known for Myxobacterial surface motility control (74, 71). Reading across to extensive gliding research on Myxobacteria (75) implies that four full gliding machines could be produced from the *B. bacteriovorus* operons, although it is also possible that some gliding proteins could encode transporters (76, 77). Their differential expression during entering, inside, exiting, and outside prey bacteria suggests that a specific engine may be required to operate in the physiologically and chemically different environments encountered at those points. Whether different gliding engines are built at different predation stages is yet to be determined experimentally. Something related was also found for flagellar rotation (powered by ions passing through a MotAB protein complex), where three pairs of different *B. bacteriovorus motAB* genes are differentially expressed entering/preparing to exit/exiting prey (65). In this case, the different motors would power one propeller type, allowing adaptation to different ionic environments during different predatory lifecycle stages.

After flagellar and/or gliding motility brings *B. bacteriovorus* to the prey surface, the flagellum is no longer involved (in invasion), but a gliding engine or type IV pilus retraction could aid in inward movement. This concept is challenging to reconcile against the osmotic pressure of the live prey cell (as discussed later). Kaplan and colleagues (78) showed that the flagellum is actually retracted into the periplasm of the *B. bacteriovorus* itself during entry to prey, in an act of economic protein recycling. A new flagellum (23, 54) or a gliding engine is built later for prey exit (71), depending on the liquidity of the external environment, but how this is sensed/anticipated is yet to be discovered.

No single required receptor on prey has been found for *B. bacteriovorus* HD100 predator attachment and invasion, nor should it be, given the variety of gram-negative prey encountered (79, 80). Some predator strains are reported to have a tighter specificity for prey type (81), and studies on specificity in those strains will be needed to reveal more about those mechanisms. Some studies, in recent years by the Mitchell and Selan labs, report reductions of gram-positive bacterial numbers in biofilms and cultures by the action of *B. bacteriovorus* (82, 83). It is understood that hydrolytic enzymes of *Bdellovibrio* could act on some gram-positive cells if they attach to them or if the *Bdellovibrio* themselves lyse near to them, releasing such enzymes. Predatory invasion into gram-positive cells is not thought to occur, but ongoing debate remains in the field about mechanisms of gram-positive cell killing and how they overlap with gram-negative predation by *B. bacteriovorus*. The periplasm of gram-negative cells makes an outer membrane-bound, stabilized environment in which sequential and exhaustive degradation of prey components by invasive predators occurs, allowing replication of several predators per prey. Gram-positive bacteria lack this environment.

Recognition was found by Varon and Shilo (25) to be of components of the outer membrane of gram-negative prey itself, including the O antigen, with recognition implicated to be by multiple *B. bacteriovorus* surface structures. In early work, Susan Koval brought a first view of prey cell defense against predation by *B. bacteriovorus*, showing that expressing a surface proteinaceous S-layer reduced predation by obstructing the prey surface, although polysaccharide capsules on prey did not (84, 85). This view was enhanced by recent work from the Whiteley Lab, which showed that expression of numerous surface curli fimbriae also defended against predation (86). These surface features cover many of the prey outer membrane regions at which *B. bacteriovorus* could recognize, attach, and invade. This is a rare case where a similar mechanism can apply to resistance against phage attacking bacterial biofilms (87). Importantly, changes in outer membrane composition of prey bacteria, including loss of OmpF porins, discovered by Mun, Mitchell and colleagues (88), and modification of outer surface coverage or outer membrane content, discovered by Ledvina, Sayegh, Whiteley and colleagues (86, 89), confer a significant level of prey defense against predation.

Components of the chemically complex gram-negative outer membrane of prey must be recognized for *B. bacteriovorus* attachment and invasion. Factors beyond Type IV pili are certainly used in prey recognition. A large family of mosaic adhesive trimer (MAT) fiber proteins were discovered to be expressed on the surface of *B. bacteriovorus*, each with different fiber tips that could bind to diverse surface molecules on the prey (90). The extent and diversity of these structures allow for the recognition of prey bacteria with different outer membrane chemistries, and MAT composition could be seen to vary between different predator strains. Their discovery dovetails with fibers seen at the prey interaction pole of *B. bacteriovorus* in early and recent microscopic work (48, 78, 91).

Invasion into the prey periplasm has remained complex and elusive to chemically define even 60 years on, its study perhaps hindered by its rapid, transient nature and the challenge of tightly synchronizing *Bdellovibrio* predator-prey cultures. Invasion by the stationary predator itself is a multifactorial process, following initial safe attachment to the prey with recognition and safe detachment for entry via forming a pore in the prey outer membrane and wall (92, 93), which is strong enough to resist the outward osmotic pressure of the live prey cell. One of the best early images of invasion was taken, using thin section electron microscopy, by Burnham and colleagues (49). It shows an intriguing bubble of outer membrane forming at the predator invasion point. The bubble contains prey outer membrane and wall, and localized damage to prey cell wall peptidoglycan and displacement of the cytoplasmic membrane is also seen when the *B. bacteriovorus* begins to enter the inner prey periplasm. Much later, Burnham’s bubble structure was shown to contain *B. bacteriovorus* CpoB (a regulator of peptidoglycan synthetic enzyme PBP1B) and also several MAT fiber proteins that participate in prey invasion processes (90). The bubble remains deposited in the outer layer of invaded prey, plugging the site of invasion after predator entry. This structure may also provide a means for escape later in the predatory cycle; further work is required to prove this, but it is suggested by observations that *B. bacteriovorus* has more than one way to exit the prey cell wall. This is either by wholesale hydrolysis or through a pre-formed pore, which we hypothesize may be the vesicle deposition site (94–96).

In more excellent electron microscopy, Burnham, Scherff, and colleagues found a round structure on the invasive predatory pole of the *B. bacteriovorus*, which Burnham called a “mesosome” and Scherff an “infection cushion,” and later, Kaplan and colleagues showed it, in much more detail, to have a complex “rose-like” structure (49, 78, 92). This predator structure was located above a circular collar structure formed in the prey outer membrane and wall, at the predator-prey interface; the *B. bacteriovorus* could deform and become thinner to enter through this portal to the inner periplasm between prey wall and cytoplasmic membrane (92, 97). In regard to predator deformation/pliability, it will be interesting to see if *Bdellovibrio* peptidoglycan is as porous as that of Myxococci, allowing for motor engines to deform it (98). The *B. bacteriovorus* cell “comma-shape” may contribute a pivoting ability to periplasmic invasion, as Banks and colleagues showed that an LD-carboxypeptidase self-wall modification mutant (abolishing the comma shape to a more straightened form) invaded prey more slowly (9). The collar, deposited in the prey, was shown, by Kuru and colleagues, using fluorescent D-amino acid labeling, to be produced by the *B. bacteriovorus*, building a reinforced molecular porthole in the prey peptidoglycan at the contact point between predator and prey that is later plugged with D-amino acid containing material once the predator is inside (99). Cryo-electron microscopy by Kaplan and colleagues (78) showed that this collar structure can expand during predator entry, accommodating the invading predator (whose cells are seen in deformed shapes), before being sealed after it enters. The collar effect and the deformation of the predator, “squeezing” through it, combine to produce an effect that likely minimizes loss of prey cell content during invasion.

The invasion complex “rose structure” on the *B. bacteriovorus* piliated cell pole is likely to be composed of many proteins because invasion occurs through the different chemical entities of the prey outer membrane and cell wall. Further work will reveal all the contents of this structure. A clue to one invasion protein came from the work of Tyson and colleagues, who found, by fluorescent detection and microscopy, a MIDAS adhesin protein at the invasion contact point between predator and prey. When the MIDAS protein was deleted, a percentage of the *B. bacteriovorus* failed to invade, and some empty (no internal predator) but killed prey, were produced by predation with this MIDAS mutant, containing known early enzymes of predation (100). This suggested that without the MIDAS adhesin, some predators may fall off prey during predatory invasion and killing. Only a percentage did this, reflecting the multifactorial nature of predator attachment to prey during invasion. More work is required to get the full picture of invasion, which occurs rapidly, approximately 15–30 min after prey contact, taking only 5–10 min to complete. Before active movement inward, many localized lipid and wall-lytic enzymes and processes must be required to get the predator tightly through the outer membrane and wall of prey bacteria without them breaking or leaking. Despite 60 years of research, many of these invasive processes are yet to be explained and offer scientific adventures ahead.

*B. bacteriovorus* kills *E. coli* prey some 20–25 min after entry and modifies prey cell wall and shape but goes on living within the prey periplasm without breaking the prey cell envelope for a further 3.5–4 h until programmed exit occurs (24, 31). Later work showed that *E. coli* prey transcription ceased around 20–25 min after *B. bacteriovorus* invasion (101). These studies changed some of the early language used to describe *B. bacteriovorus*, nullifying early description of it as a parasite, instead informing us that, as a true, endobiotic predator, it kills and modifies a dead cell to become a temporary stable home.

Building that home also involves keeping the cytoplasmic membrane of the prey cell intact (95), and this must be robust enough to allow for the degradation of inner DNA, RNA, proteins, and carbohydrates that are used by the growing and replicating predator from within that barrier. A picture of the invasion, killing, growth, and exit from prey was developed by early researchers (17, 20, 23, 26, 32, 39, 102–104). This complex sequential lifecycle was hard to study as there were discrete functional stages of little-studied processes such as prey periplasm modification for bdelloplast stabilization and discrete waves of regulated hydrolysis so that proteases didn’t consume nucleases, for example. Excellent early (105) sampling and purification of proteins from bdelloplasts, by Ruby and colleagues, at timepoints during the predatory cycle, identified some diverse predatory enzymes. Some were found to degrade prey DNA and RNA components for reuse as monomers by the predator cells (45, 106). Some act on prey cell walls; Fackrell and Robinson (107) purified and characterized a “lytic protease” from *Bdellovibrio* in the 1970s, showing that it acted upon peptidoglycan peptides, and Tudor, Thomashow, and colleagues discovered peptidoglycan deacetylation processes (38, 108) and prey cell wall modifications (37). Thomashow, Ruby, and colleagues discovered a peptidoglycan lytic enzyme mixture from *B. bacteriovorus* during predation, Ruby using it to prematurely release predators during predation and study their biology (36, 109). They inspired the later discovery of a specific prey exit lysozyme by Chris Harding, Simona Huwiler and colleagues (discussed later) (96).

Replication of predator inside prey was found by Shilo and Martin Kessel to be via multi-nucleoid growth of *B. bacteriovorus* as a filament and its later fission into progeny, a significant contrast to more usual bacterial growth by binary fission. This novel *B. bacteriovorus* growth mode allows for odd- and even-numbered progeny, allowing maximum predator numbers to be yielded from the finite resources contained in a single prey cell (23, 78, 94, 104, 110). The small size of the *B. bacteriovorus* cell at approximately 0.3 × 1.0 µm versus the large size of its genome at 3.85 Mb brought with it a need for tight chromosome packing, involving folding at some 450 sites on the predator chromosome by a histone-like protein, and a cellular organization situating ribosomes around the periphery of a large nucleoid (78, 111–115).

There are several challenges posed by the unusual division mode and the need to position daughter chromosomes at different locations in the dividing filament, relative to progeny number. The multinucleoid nature of predatory replication and its synchronization has been studied by the labs of Jolanta Zakzewska-Czerwinska and Geraldine Laloux, who have also reviewed predator cell division processes (116, 117). Makoswki and colleagues labeled DNA-replisome protein DnaN with an mNeon tag, finding that DNA replication and chromosome segregation were completed in advance of the long, multi-nucleoid *B. bacteriovorus* filament septating (118, 119). Kaljevic and colleagues (115) monitored and quantified replication dynamics using a natively expressed fluorescent DnaN fusion. Progressive chromosome segregation during filamentous growth was shown in the same publication using ParB fluorescent fusions and direct labeling of *oriC* and *ter* loci (which also revealed the polar, longitudinal organization of the chromosome).

Progeny *B. bacteriovorus* differentiate to have a predatory pole and, if the bdelloplast is in a liquid environment, a flagellar pole, before prey exit. Milner, Lowry, and colleagues defined that specifying the invasive pole in predatory *B. bacteriovorus* required the GTP-binding protein MglA, and that this polarity also involved RomR; both proteins are known components regulating polarity in the gliding pack hunting bacteria *Myxococcus xanthus* (74, 75, 120). Deletion of *mglA* rendered *B. bacteriovorus* HD100 non-predatory. Milner, Ray, and colleagues demonstrated that three ParA homologues of chromosome-partitioning proteins are encoded by *B. bacteriovorus*. These include one solo ParA, which regulates gliding motility, another of unknown function, and a canonical ParAB, from an operon (adjacent to *divIVA*), which is involved in pole specification and sporulation in gram-positive bacteria (121). Regulation of this canonical ParAB was further studied by Kaljevic and colleagues (122). Remy and colleagues (123) discovered that RomR and DivIVA participate in marking poles prior to *B. bacteriovorus* filament division, showing DivIVA dynamically moving from pole to pole in non-replicating *B. bacteriovorus* and RomR marking future invasive poles. Using biotin-dependent proximity labeling, they showed that RomR interacts with known invasive pole proteins, and this technique may allow future protein-protein interaction understanding inside *B. bacteriovorus* (123).

The novel replication and septation of *B. bacteriovorus* brought experimental issues of obtaining faithful plasmid segregation and copy number along the long filament when trying to achieve genetic manipulation. Thus, genetic studies of *B. bacteriovorus* were initially aided by the conventional use of large insert genomic libraries (61), coupled with single-gene deletions using kanamycin resistance markers. Transposon mutagenesis was also employed (124, 125) in prey-independent (host-independent [HI]) strains, and although successful, in such a complex process as predation, with many novel *B. bacteriovorus* genes, it was hard in the early years to define function for many predation-defective mutants. Gene defects were, however, later systematically analyzed and grouped into predation stages to aid discovery of their functions (126).

Forward bacterial genetics, deletion, and complementation of genes to discern function were relatively slow to take off in *B. bacteriovorus*. Conventional IncQ, RK2, and derived plasmids used for complementation tests in diverse gram-negative soil bacteria did not replicate in *B. bacteriovorus*. In contrast to other gram-negative bacteria, the range of antibiotic resistance markers is limited in *B. bacteriovorus*, likely due in part to its novel changes to cell wall and membrane biochemistry. Kanamycin resistance (via neomycin phosphotransferase) was one of the few selectable markers in *B. bacteriovorus*, thus limiting the sophistication of early gene manipulation experiments.

However, somewhat counterintuitively for an *E. coli*-killing predator, IncP plasmids allowed delivery into *B. bacteriovorus* via conjugation, using the RP4 origin for conjugal transfer, from *E. coli* donors. This worked by physically flooding the predators (which invade *E. coli* using only their non-flagellar pole) with many plasmid donors, all allowing them to occupy other positions around the predator cell where conjugation and gene transfer could occur. Todd Cotter, Michael Thomashow, and Silvia Pinero (127, 128) were instrumental in this work, which was used and developed further in predatory gene function with fluorescent gene product tagging. Although the IncP plasmids didn’t replicate in *B. bacteriovorus*, they allowed recombination and with counter selection against the plasmid and allelic exchange for gene replacement, gene deletion, or introduction of point-mutated or fluorescently tagged gene products. This enabled studies of predatory protein function and cellular or bdelloplast location during predation (90, 129–131). This deepened the understanding of excellent biochemistry and cell biology studies done in the 1960s and 1970s and also revealed some of the gene regulation that underpinned them during the predatory cycle. Later, an engineered RSF1010 replicon was used successfully to express fluorescent protein gene *tdtomato* from a plasmid in *B. bacteriovorus*, using a neomycin phosphotransferase promoter and gentamicin selection; this added to possibilities for *B. bacteriovorus* gene manipulation (132).

A fact that makes *B. bacteriovorus* so useful experimentally is that they can be cultured in a prey-independent manner, allowing the test deletion of genes postulated to be required for the predatory lifecycle with subsequent rescue and testing of mutants. *B. bacteriovorus* is sometimes slightly confusingly described as obligately predatory, but Shilo and Reiner (30) discovered that predatory *B. bacteriovorus* can convert to grow “host”/prey-independently on media rich in proteins and vitamins containing peptone and yeast extract. Spontaneously occurring mutations occur naturally in predatory populations (1 in 10^7^ in populations of approximately 10^10^ predatory cells per mL) and were found (discussed below) to convert predators to prey-independent axenic growth on such media (133). Although such prey or host-independent (HI) *B. bacteriovorus* HD100 may be less rapidly motile and have a longer cell length than the predatory form, they can invade prey cells. So, if a directed gene mutation is introduced to a wild-type HI strain of predator, then comparative predation experiments can be carried out with the deletion HI strain versus the wild-type HI strain (68, 134). Genome sequencing allows a check against any other point mutations in such comparisons.

Cotter and Thomashow (133) worked on prey-independent *B. bacteriovorus* in the 1990s and first discovered genetic loci, at a “host-independence” “hit” locus, including novel genes *bd0108* and *bd0109*, which could convert *B. bacteriovorus* to prey-independent growth. Capeness and colleagues (135) showed that some genes at the locus link Type IV pilus activity to the onset of growth, a checkpoint that may occur after prey invasion or when the genes themselves were mutated. Roschanski and colleagues (136) discovered that RhlB, an RNA helicase encoded at the *hit* locus, is important for axenic growth.

Barel and Jurkevitch (137) and Medina and Kadouri (138) further characterized the novel phenotypes of prey-independent *B. bacteriovorus*. They and Shilo (30) showed that in the absence of prey, *B. bacteriovorus* grew in amorphous cell shapes with uneven division planes and that they could form biofilms and could divide to reform predatory cells. The prey-independent *B. bacteriovorus* are elongated with diverse morphology (103) and not so highly motile as attack phase cells but can also retain predatory capacity if presented with prey. This has allowed comparisons of predatory ability where a gene deletion may severely affect predation, but a culture can be “saved” prey independently and the phenotype examined (55, 68).

*B. bacteriovorus* clearly has many processes to differentially control to achieve prey invasion versus prey-independent growth, with some environmental species running both lifestyles simultaneously (139). Analysis by Galperin of cyclic-di-GMP signaling genes in the *B. bacteriovorus* genome, versus many other bacterial genomes, showed a relative abundance of signaling pathways (140). Gene deletion studies of cyclic-di-GMP synthases, as well as fluorescent tagging to determine their cellular addresses and timing of expression, by Hobley and colleagues showed that cyclic-di-GMP can signal whether *B. bacteriovorus* adopts a predatory or non-predatory lifestyle (141). Later, it was found that cyclic GAMP signaling was also involved in the regulation of prey exit (142). Jurkevitch and Jenal identified 84 cyclic-di-GMP binding proteins, including some secretion proteins or enzymes of central metabolism, indicating the importance of such signaling in both predatory and non-predatory growth (143). A study, by Sathyamoorthy and colleagues, of two cyclic-di-GMP receptors in the PilZ family, which can act as flagellar brakes, revealed that braking of the flagellum by cyclic-di-GMP can signal to a non-predatory lifestyle (144). Meek and Lovering characterized the mechanism by which an invasion-associated cyclic-di-GMP synthase can be activated; this implicated it as part of a wider phosphorylation cascade that remains to be fully studied (145).

Despite the challenges posed by the staged lifecycle, several labs have made progress in understanding regulatory processes therein. Careful microbiological culturing with near-synchronous predatory prey invasion (146), plus RNA sampling, allowed transcriptional studies of gene expression across the predatory cycle. This enhanced understanding of timing of biochemical processes and how and when they were required for predation to initiate, proceed, and complete. Lambert and colleagues (72) examined transcriptional changes occurring upon prey invasion (the “predatosome”), sparking further research in processes upregulated (prey peptidoglycan remodeling and gliding motility) or downregulated (flagellar genes). Jurkevitch lab members assessed proteomic and transcriptomic changes during stages of predatory and non-predatory growth, discovering many functional and regulatory processes in predation. To give just some examples, Karunker, Rotem, and colleagues discovered that the FliA flagellar sigma factor, normally used in flagellar gene upregulation, was used after prey entry to control some non-flagellar, predatory genes (147). Barel and colleagues (146) tracked prey and predator outer membrane proteins during predation, detecting predator-driven cleavage of prey outer membrane protein(s) (OMPs), and Dori-Bachash and colleagues mapped an initial proteomic landscape further defining many functions specific to predatory or prey-independent growth (148). In 2025, this was followed in the Huwiler lab with more modern proteomic methods, discovering the timing of expression of 61% of *B. bacteriovorus* predatory proteins in tight succession - gliding proteins and proteolytic enzymes among other predatory processes expressed across predation (73). This group has also derived new methods to quantify specific protein secretion from *B. bacteriovorus* (149).

Recently, both the Jurkevitch lab and Laloux lab have established CRISPRi, and the latter also IPTG-inducible expression, to control gene expression and mediate gene interference techniques allowing gene depletion at specific points during predation (144, 150). FtsZ depletion caused incomplete division of *B. bacteriovorus* cell filaments inside prey with abnormally long *B. bacteriovorus* cells exiting prey and showing that septation was not a signal prior to prey exit (150). More experiments of this kind will allow a more sophisticated interrogation of predation processes rather than the deletion of gene function.

While *B. bacteriovorus* is growing for several hours within the bdelloplast, important modifications ensure that the prey cell wall and membrane layers are strong enough to resist the pressures of prey nutrient depletion and predator growth and replication within. Studying genes whose transcription was detected at the start of predation (72), Lambert, Lerner and colleagues showed that *B. bacteriovorus* could deacetylate prey cell wall peptidoglycan at GlcNAc residues by secreting two enzymes into the prey periplasm (95). They also showed that D-amino acid peptide cross-links in prey cell walls were modified by two *Bdellovibrio* DacB enzymes at this time and that this converted rod-shaped prey bacteria to robust but spherical bdelloplasts containing a growing predator. Interestingly, if the *dacB* genes were deleted, then multiple occupancy of prey bacteria became more common, whereas single invasion is the norm. This shows that early prey cell wall modification across the bdelloplast serves as a signal to subsequent *B. bacteriovorus* to prevent superinfection (151). These DacB initial modifications may also have made those cross-links available to other predator enzymes, such as LD-transpeptidases, for further modification to strengthen prey-wall cross-links.

Kuru and colleagues showed microscopically that (fluorescently labeled) D-amino acids were added across the whole of the prey bdelloplast wall by predatory LD-transpeptidase enzymes, with the wave of likely strengthening modification spreading from the predatory entry point across the prey cell as it rounded to a bdelloplast upon predator invasion (99). In another predator action, Banks and colleagues found that *B. bacteriovorus* could produce an MltA protein that acted on the specific septal peptidoglycan of any bdelloplasts made from dividing prey cells. This expands the available compartment size in which the predator could replicate allowing the predator to cross from one side to the other of a dividing prey cell, “walking the plank” to access nutrients on both sides of the septal divide (152).

Together, these peptidoglycan modifications make the dead prey cell into a spacious, strengthened home that accommodates the predator growth within. Examining bdelloplasts made by predators deleted for prey-GlcNAc deacetylation also showed that access into the inner periplasm, (prey cell wall-inner membrane compartment), is the usual prey site where *B. bacteriovorus* resides, with the strengthened prey cell wall “outside” of it. Mutants failing in deacetylation often failed to cross the wall and thus they live, (by possibly compromised predatory growth), between the prey wall and outer membrane instead (95). This result, and the suggestions from subsequent work on a MIDAS adhesion mutant (100), suggest that prey inner membrane contact may not be absolutely required for prey killing, even though predators certainly modify prey cytoplasmic membranes during predation.

Addressing issues of self-preservation while killing another bacterium made of similar materials led Cadby and colleagues to ask “how does a predator’s cell wall-damaging enzyme bypass damaging the cell wall of *B. bacteriovorus* as it is secreted from its periplasm into the periplasm of the prey bacterium?”. They identified a potential ankyrin immunity protein gene encoded downstream of one of two separate *dacB* genes. Lambert examined experimentally the phenotype of derived mutants (134). Together, they found that the ankyrin-deleted mutant could be derived and cultured prey independently, but when presented with prey bacteria, it attached to the prey; instead of rounding them up to make a bdelloplast, it rounded up and killed itself instead. The multiplicity of ankyrin proteins (and those of other toxin immunity protein folds) encoded by the *B. bacteriovorus* genome hints that additional mechanisms against other effectors/toxins remain to be studied.

Another example of predator protein-protein systems, in the *B. bacteriovorus* orchestration of cell wall modification and damage, followed from the peptidoglycan GlcNAc deacetylase studies. This work proposed that waves of prey cell wall deacetylation encoded by the peptidoglycan deacetylases (95) protected the bdelloplast from premature bursting, by prey- or predator-conventional lysozymes, which do not cut deacetylated “irregular” peptidoglycan. Several lysozymes are encoded in the *B. bacteriovorus* genome and were being studied genetically for function. However, Lovering and colleagues spotted that one of these lysozymes had an abnormal peptidoglycan binding site. Harding expressed and determined the structure of the unusual DslA lysozyme protein, and Huwiler was able to show that DslA could cut the deacetylated peptidoglycan of prey bdelloplasts and that without the *dslA* gene, *B. bacteriovorus* were slow to exit the prey cells (96). Again, this slowness, rather than a null phenotype, suggested that enzymes additional to DslA can surpass the need for a single enzyme for exit. There are several exit options for the predator to break prey bdelloplast peptidoglycan with different enzymes or to damage the prey outer membrane with others. To use an English phrase, a belt and braces (belt and suspenders) evolutionary approach is employed by *B. bacteriovorus*. They use duplication and/or diversification to have more than one mechanism/enzyme to achieve important outcomes such as exit. These act at many key points in predation where predators might otherwise get “stuck” inside a dead prey cell if a sole gene product for a process failed.

Although some cell wall modifications of prey by predator have been the subject of successful studies to reveal some mechanisms, there is much more to learn. For instance, the nature of *B. bacteriovorus* outer membrane to prey outer membrane encounter during recognition/invasion, the entry of predator through prey outer membrane (without getting stuck), the deposition of the bubble or vesicle of membrane at the invasion point, and the OM remaining on predators inside prey still hold mysteries. The membrane-sheathing of the flagellum (49) may prevent the flagellum protein filament snagging on the outside of the prey OM while the flagellum is shortening (54) during flagellar retraction into the predator periplasm (78). How the predator and prey outer membranes resolve during invasion is still unknown mechanistically. The two outer membranes are challenging to resolve biochemically while *B. bacteriovorus* is inside its prey. Nelson and Rittenberg began in the 1980s to characterize the lipid A of predator outer membrane in contrast to that of *E. coli* prey, reporting that prey lipid A was incorporated into predator outer membrane during predatory growth (153, 154). Schwudke and colleagues (155) showed that novel mannosylation of the Lipid A of *B. bacteriovorus* outer membranes occurs, replacing phosphate groups and distinguishing them from more usual gram-negative lipid As. This may allow predatory enzymes to attack prey lipid A, for entry and exit, without predator damage, but much more exciting detail awaits new discoveries.

Much more is to be learned about the chemistry of the *B. bacteriovorus* outer membrane, but Parr and colleagues (156) have already shown that *B. bacteriovorus* outer membrane proteins can be unusual in form, such as a pentameric porin-like protein recently discovered.

What is the grand sum of what we have learned about this bacterial predator? It is certainly clear that the complexity and organization of its enzymes for prey modification are many, and this makes it an interesting model system for further experimentation and for applied exploitation. If there is one outcome to center on, it is that necessity is the mother of invention. The requirement to devour another bacterium, from within, with the utmost economy, has afforded *Bdellovibrio bacteriovorus* a variety of fail-safe mechanisms, with back-ups, that simply (once discovered!) “make sense.” With all the new techniques available, we await the next swathe of these and what they can tell us about both the “flagship” and the “pirates.”

## References

[1] Stolp H, Petzold H. 1962. Untersuchungen über einen obligat parasitischen Mikroorganismus mit lytischer Aktivität für *Pseudomonas*-Bakterien. J Phytopathol 45:364–390. doi:10.1111/j.1439-0434.1962.tb02050.x

[2] Stolp H. 1973. The bdellovibrios: bacterial parasites of bacteria. Annu Rev Phytopathol 11:53–76. doi:10.1146/annurev.py.11.090173.000413

[3] Stolp H, Starr MP. 1963. *Bdellovibrio bacteriovorus* gen. et sp. n., a predatory, ectoparasitic, and bacteriolytic microorganism. Antonie Van Leeuwenhoek 29:217–248. doi:10.1007/BF02046064 14068454

[4] Rendulic S, Jagtap P, Rosinus A, Eppinger M, Baar C, Lanz C, Keller H, Lambert C, Evans KJ, Goesmann A, et al.. 2004. A predator unmasked: life cycle of *Bdellovibrio bacteriovorus* from a genomic perspective. Science 303:689–692. doi:10.1126/science.1093027 14752164

[5] Cavalier-Smith T. 2002. The neomuran origin of archaebacteria, the negibacterial root of the universal tree and bacterial megaclassification. Int J Syst Evol Microbiol 52:7–76. doi:10.1099/00207713-52-1-7 11837318

[6] Wurzel O, Dori-Bachash M, Pietrokovski S. 2010. Mutation detection with next generation resequencing through a mediator genome. PLoS One 5:e15628. doi:10.1128/journal.pone.0015628 PMC3013116 21209874

[7] Oren A, Garrity GM. 2021. Valid publication of the names of forty-two phyla of prokaryotes. Int J Syst Evol Microbiol 71. doi:10.1099/ijsem.0.005056 34694987

[8] Hoshiko Y, Okuno M, Yamamoto T, Maeda T, Ogura Y. 2024. Improved complete genome sequence of *Bdellovibrio bacteriovorus* 109J, a widely studied laboratory strain of predatory bacteria. Microbiol Resour Announc 13:e0129623. doi:10.1128/mra.01296-23 PMC11256856 38847525

[9] Banks EJ, Valdivia-Delgado M, Biboy J, Wilson A, Cadby IT, Vollmer W, Lambert C, Lovering AL, Sockett RE. 2022. Asymmetric peptidoglycan editing generates cell curvature in *Bdellovibrio* predatory bacteria. Nat Commun 13:1509. doi:10.1038/s41467-022-29007-y PMC8938487 35314810

[10] Williams HN, Kelley JI, Baer ML, Turng B-F. 1995. The association of bdellovibrios with surfaces in the aquatic environment. Can J Microbiol 41:1142–1147. doi:10.1139/m95-159

[11] Williams LE, Cullen N, DeGiorgis JA, Martinez KJ, Mellone J, Oser M, Wang J, Zhang Y. 2019. Variation in genome content and predatory phenotypes between *Bdellovibrio* sp. NC01 isolated from soil and *B. bacteriovorus* type strain HD100. Microbiology (Reading) 165:1315–1330. doi:10.1099/mic.0.000861 PMC7137782 31592759

[12] Williams HN, Chen H. 2020. Environmental regulation of the distribution and ecology of *Bdellovibrio* and like organisms. Front Microbiol 11:545070. doi:10.3389/fmicb.2020.545070 PMC7658600 33193128

[13] Gadkari D, Stolp H. 1976. Energy metabolism of *Bdellovibrio bacteriovorus*. II. P/O ratio and ATP pool turnover rate. Arch Microbiol 108:125–132. doi:10.1007/BF00425102 179488

[14] Gadkari D, Stolp H. 1975. Energy metabolism of *Bdellovibrio bacteriovorus*. I. Energy production, ATP pool, energy charge. Arch Microbiol 102:179–185. doi:10.1007/BF00428366 1156083

[15] Seidler RJ, Starr MP. 1968. Structure of the flagellum of *Bdellovibrio bacteriovorus*. J Bacteriol 95:1952–1955. doi:10.1128/jb.95.5.1952-1955.1968 PMC252230 5650092

[16] Seidler RJ, Starr MP. 1969. Isolation and characterization of host-independent bdellovibrios. J Bacteriol 100:769–785. doi:10.1128/jb.100.2.769-785.1969 PMC250157 4901359

[17] Seidler RJ, Starr MP. 1969. Factors affecting the intracellular parasitic growth of *Bdellovibrio bacteriovorus* developing within *Escherichia coli*. J Bacteriol 97:912–923. doi:10.1128/jb.97.2.912-923.1969 PMC249777 4886299

[18] Snellen JE, Starr MP. 1976. Alterations in the cell wall of *Spirillum serpens* VHL early in its association with *Bdellovibrio bacteriovorus* 109D. Arch Microbiol 108:55–64. doi:10.1007/BF00425093 818972

[19] Snellen JE, Starr MP. 1974. Ultrastructural aspects of localized membrane damage in *Spirillum serpens* VHL early in its association with *Bdellovibrio bacteriovorus* 109D. Arch Microbiol 100:179–195. doi:10.1007/BF00446316 4615643

[20] Starr MP, Baigent NL. 1966. Parasitic interaction of *Bdellovibrio bacteriovorus* with other bacteria. J Bacteriol 91:2006–2017. doi:10.1128/jb.91.5.2006-2017.1966 PMC316158 5327913

[21] Jurkevitch E. 2006. A brief history of short bacteria: a chronicle of *Bdellovibrio* (and like organisms) research. *In* Predatory prokaryotes. Microbiology monographs, 1st ed. Springer-Verlag, Berlin Germany.

[22] Shilo M, Bruff B. 1965. Lysis of gram-negative bacteria by host-independent ectoparasitic *Bdellovibrio bacteriovorus* isolates. J Gen Microbiol 40:317–328. doi:10.1099/00221287-40-3-317 5325037

[23] Shilo M. 1969. Morphological and physiological aspects of the interaction of *Bdellovibrio* with host bacteria. Curr Top Microbiol Immunol 50:174–204. doi:10.1007/978-3-642-46169-9_6 4907693

[24] Varon M, Drucker I, Shilo M. 1969. Early effects of *Bdellovibrio* infection on the syntheses of protein and RNA of host bacteria. Biochem Biophys Res Commun 37:518–525. doi:10.1016/0006-291X(69)90946-2 4900142

[25] Varon M, Shilo M. 1969. Attachment of *Bdellovibrio bacteriovorus* to cell wall mutants of *Salmonella* spp. and *Escherichia coli*. J Bacteriol 97:977–979. doi:10.1128/jb.97.2.977-979.1969 PMC249794 4886305

[26] Varon M. 1968. Interaction of *Bdellovibrio bacteriovorus* and host bacteria. J Bacteriol 95:744–753. doi:10.1128/jb.95.3.744-753.1968 PMC252088 4868362

[27] Varon M, Shilo M. 1980. Ecology of aquatic bdellovibrios, p 1–41. *In* Droop MR, Jannesch HW (ed), Advances in aquatic microbiology. Vol. 2. Academic Press, London.

[28] Varon M, Shilo M. 1969. Interaction of *Bdellovibrio bacteriovorus* and host bacteria II. Intracellular growth and development of *Bdellovibrio bacteriovorus* in liquid cultures. J Bacteriol 99:136–141. doi:10.1128/jb.99.1.136-141.1969 PMC249978 4895842

[29] Varon M, Shilo M. 1981. Inhibition of the predatory activity of *Bdellovibrio* by various environmental pollutants. Microb Ecol 7:107–111. doi:10.1007/BF02032492 24227420

[30] Reiner AM, Shilo M. 1969. Host-independent growth of *Bdellovibrio bacteriovorus* in microbial extracts. J Gen Microbiol 59:401–410. doi:10.1099/00221287-59-3-401

[31] Rittenberg SC, Shilo M. 1970. Early host damage in the infection cycle of *Bdellovibrio bacteriovorus*. J Bacteriol 102:149–160. doi:10.1128/jb.102.1.149-160.1970 PMC284981 4908670

[32] Rittenberg S. 1982. *Bdellovibrios*-intraperiplasmic growth, p 379–394. *In* Burns RG, Slater JH (ed), Experimental Microbial Ecology. Blackwell Scientific Publications.

[33] Rittenberg SC, Langley D. 1975. Utilization of nucleoside monophosphates per Se for intraperiplasmic growth of *Bdellovibrio bacteriovorus*. J Bacteriol 121:1137–1144. doi:10.1128/jb.121.3.1137-1144.1975 PMC246046 1090594

[34] Rosson RA, Rittenberg SC. 1979. Regulated breakdown of *Escherichia coli* deoxyribonucleic acid during intraperiplasmic growth of *Bdellovibrio bacteriovorus* 109J. J Bacteriol 140:620–633. doi:10.1128/jb.140.2.620-633.1979 PMC216690 387743

[35] Hespell RB, Thomashow MF, Rittenberg SC. 1974. Changes in cell composition and viability of *Bdellovibrio bacteriovorus* during starvation. Arch Microbiol 97:313–327. doi:10.1007/BF00403070 4599992

[36] Thomashow MF, Rittenberg SC. 1978. Intraperiplasmic growth of *Bdellovibrio bacteriovorus* 109J: solubilization of *Escherichia coli* peptidoglycan. J Bacteriol 135:998–1007. doi:10.1128/jb.135.3.998-1007.1978 PMC222476 357428

[37] Thomashow MF, Rittenberg SC. 1978. Intraperiplasmic growth of *Bdellovibrio bacteriovorus* 109J: attachment of long-chain fatty acids to *Escherichia coli* peptidoglycan. J Bacteriol 135:1015–1023. doi:10.1128/jb.135.3.1015-1023.1978 PMC222478 357411

[38] Thomashow MF, Rittenberg SC. 1978. Intraperiplasmic growth of *Bdellovibrio bacteriovorus* 109J: N-deacetylation of *Escherichia coli* peptidoglycan amino sugars. J Bacteriol 135:1008–1014. doi:10.1128/jb.135.3.1008-1014.1978 PMC222477 357410

[39] Thomashow MF, Rittenberg SC. 1979. The intraperiplasmic growth cycle—the life style of the *Bdellovibrios*, p 115–136. *In* Parish JH (ed), Developmental biology of prokaryotes, 9th ed. University of California Press.

[40] Thomashow LS, Rittenberg SC. 1985. Isolation and composition of sheathed flagella from *Bdellovibrio bacteriovorus* 109J. J Bacteriol 163:1047–1054. doi:10.1128/jb.163.3.1047-1054.1985 PMC219236 4030691

[41] Thomashow LS, Rittenberg SC. 1985. Waveform analysis and structure of flagella and basal complexes from *Bdellovibrio bacteriovorus* 109J. J Bacteriol 163:1038–1046. doi:10.1128/jb.163.3.1038-1046.1985 PMC219235 4030690

[42] Hespell RB. 1976. Glycolytic and tricarboxylic acid cycle enzyme activities during intraperiplasmic growth of *Bdellovibrio bacteriovorus* on *Escherichia coli*. J Bacteriol 128:677–680. doi:10.1128/jb.128.2.677-680.1976 PMC232810 135759

[43] Hespell RB. 1978. Intraperiplasmic growth of *Bdellovibrio bacteriovorus* on heat-treated *Escherichia coli*. J Bacteriol 133:1156–1162. doi:10.1128/jb.133.3.1156-1162.1978 PMC222147 346559

[44] Hespell RB, Miozzari GF, Rittenberg SC. 1975. Ribonucleic acid destruction and synthesis during intraperiplasmic growth of *Bdellovibrio bacteriovorus*. J Bacteriol 123:481–491. doi:10.1128/jb.123.2.481-491.1975 PMC235752 1097411

[45] Hespell RB, Odelson DA. 1978. Metabolism of RNA-ribose by *Bdellovibrio bacteriovorus* during intraperiplasmic growth on *Escherichia coli*. J Bacteriol 136:936–946. doi:10.1128/jb.136.3.936-946.1978 PMC218528 363699

[46] Ruby EG, McCabe JB. 1986. An ATP transport system in the intracellular bacterium, *Bdellovibrio bacteriovorus* 109J. J Bacteriol 167:1066–1070. doi:10.1128/jb.167.3.1066-1070.1986 PMC215982 3745115

[47] Ruby EG, McCabe JB, Barke JI. 1985. Uptake of intact nucleoside monophosphates by *Bdellovibrio bacteriovorus* 109J. J Bacteriol 163:1087–1094. doi:10.1128/jb.163.3.1087-1094.1985 PMC219242 4030692

[48] Abram D, Davis BK. 1970. Structural properties and features of parasitic *Bdellovibrio bacteriovorus*. J Bacteriol 104:948–965. doi:10.1128/jb.104.2.948-965.1970 PMC285079 4099101

[49] Burnham JC, Hashimoto T, Conti SF. 1968. Electron microscopic observations on the penetration of *Bdellovibrio bacteriovorus* into gram-negative bacterial hosts. J Bacteriol 96:1366–1381. doi:10.1128/jb.96.4.1366-1381.1968 PMC252461 4879563

[50] Tudor JJ, Conti SF. 1978. Characterization of germination and activation of *Bdellovibrio* bdellocysts. J Bacteriol 133:130–138. doi:10.1128/jb.133.1.130-138.1978 PMC221986 618835

[51] Diedrich DL. 1988. Bdellovibrios: recycling, remodelling and relocalizing components from their prey. Microbiol Sci 5:100–103. 2856309

[52] Davis SC, Cerra J, Williams LE. 2024. Comparative genomics of obligate predatory bacteria belonging to phylum *Bdellovibrionota* highlights distribution and predicted functions of lineage-specific protein families. mSphere 9:e0068024. doi:10.1128/msphere.00680-24 PMC11656739 39535198

[53] Waite DW, Chuvochina M, Pelikan C, Parks DH, Yilmaz P, Wagner M, Loy A, Naganuma T, Nakai R, Whitman WB, et al.. 2020. Proposal to reclassify the proteobacterial classes Deltaproteobacteria and Oligoflexia, and the phylum Thermodesulfobacteria into four phyla reflecting major functional capabilities. Int J Syst Evol Microbiol 70:5972–6016. doi:10.1099/ijsem.0.004213 33151140

[54] Iida Y, Hobley L, Lambert C, Fenton AK, Sockett RE, Aizawa S-I. 2009. Roles of multiple flagellins in flagellar formation and flagellar growth post bdelloplast lysis in *Bdellovibrio bacteriovorus*. J Mol Biol 394:1011–1021. doi:10.1016/j.jmb.2009.10.003 PMC2791853 19819245

[55] Lambert C, Evans KJ, Till R, Hobley L, Capeness M, Rendulic S, Schuster SC, Aizawa S-I, Sockett RE. 2006. Characterizing the flagellar filament and the role of motility in bacterial prey-penetration by *Bdellovibrio bacteriovorus*. Mol Microbiol 60:274–286. doi:10.1111/j.1365-2958.2006.05081.x PMC1453311 16573680

[56] Duncan MC, Forbes JC, Nguyen Y, Shull LM, Gillette RK, Lazinski DW, Ali A, Shanks RMQ, Kadouri DE, Camilli A. 2018. *Vibrio cholerae* motility exerts drag force to impede attack by the bacterial predator *Bdellovibrio bacteriovorus*. Nat Commun 9:4757. doi:10.1038/s41467-018-07245-3 PMC6232129 30420597

[57] LaMarre AG, Straley SC, Conti SF. 1977. Chemotaxis toward amino acids by *Bdellovibrio bacteriovorus*. J Bacteriol 131:201–207. doi:10.1128/jb.131.1.201-207.1977 PMC235410 17594

[58] Straley SC, Conti SF. 1977. Chemotaxis by *Bdellovibrio bacteriovorus* toward prey. J Bacteriol 132:628–640. doi:10.1128/jb.132.2.628-640.1977 PMC221905 410796

[59] Straley SC, Conti SF. 1974. Chemotaxis in *Bdellovibrio bacteriovorus*. J Bacteriol 120:549–551. doi:10.1128/jb.120.1.549-551.1974 PMC245800 4608875

[60] Straley SC, LaMarre AG, Lawrence LJ, Conti SF. 1979. Chemotaxis of *Bdellovibrio bacteriovorus* toward pure compounds. J Bacteriol 140:634–642. doi:10.1128/jb.140.2.634-642.1979 PMC216691 500565

[61] Lambert C, Smith MCM, Sockett RE. 2003. A novel assay to monitor predator–prey interactions for *Bdellovibrio bacteriovorus* 109 J reveals a role for methyl-accepting chemotaxis proteins in predation. Environ Microbiol 5:127–132. doi:10.1046/j.1462-2920.2003.00385.x 12558595

[62] Berg HC. 1975. Bacterial behaviour. Nature 254:389–392. doi:10.1038/254389a0 1090851

[63] Berg HC. 1975. Chemotaxis in bacteria. Annu Rev Biophys Bioeng 4:119–136. doi:10.1146/annurev.bb.04.060175.001003 1098551

[64] Berg HC, Purcell EM. 1977. Physics of chemoreception. Biophys J 20:193–219. doi:10.1016/S0006-3495(77)85544-6 PMC1473391 911982

[65] Morehouse KA, Hobley L, Capeness M, Sockett RE. 2011. Three *motAB* stator gene products in *Bdellovibrio bacteriovorus* contribute to motility of a single flagellum during predatory and prey-independent growth. J Bacteriol 193:932–943. doi:10.1128/JB.00941-10 PMC3028683 21148728

[66] Im H, Kwon H, Cho G, Kwon J, Choi SY, Mitchell RJ. 2019. Viscosity has dichotomous effects on *Bdellovibrio bacteriovorus* HD100 predation. Environ Microbiol 21:4675–4684. doi:10.1111/1462-2920.14799 31498968

[67] Mahmoud KK, Koval SF. 2010. Characterization of type IV pili in the life cycle of the predator bacterium *Bdellovibrio*. Microbiology (Reading) 156:1040–1051. doi:10.1099/mic.0.036137-0 20056705

[68] Evans KJ, Lambert C, Sockett RE. 2007. Predation by *Bdellovibrio bacteriovorus* HD100 requires type IV pili. J Bacteriol 189:4850–4859. doi:10.1128/JB.01942-06 PMC1913455 17416646

[69] Talà L, Fineberg A, Kukura P, Persat A. 2019. *Pseudomonas aeruginosa* orchestrates twitching motility by sequential control of type IV pili movements. Nat Microbiol 4:774–780. doi:10.1038/s41564-019-0378-9 PMC6522360 30804544

[70] Kadouri D, O’Toole GA. 2005. Susceptibility of biofilms to *Bdellovibrio bacteriovorus* attack. Appl Environ Microbiol 71:4044–4051. doi:10.1128/AEM.71.7.4044-4051.2005 PMC1169041 16000819

[71] Lambert C, Fenton AK, Hobley L, Sockett RE. 2011. Predatory *Bdellovibrio* bacteria use gliding motility to scout for prey on surfaces. J Bacteriol 193:3139–3141. doi:10.1128/JB.00224-11 PMC3133215 21515772

[72] Lambert C, Chang CY, Capeness MJ, Sockett RE. 2010. The first bite— profiling the predatosome in the bacterial pathogen *Bdellovibrio*. PLoS One 5:e8599. doi:10.1371/journal.pone.0008599 PMC2797640 20062540

[73] Lai TF, Jankov D, Grossmann J, Roschitzki B, Huwiler SG. 2025. Quantitative proteome of bacterial periplasmic predation by *Bdellovibrio bacteriovorus* reveals a prey-lytic protease. Commun Biol 8:1491. doi:10.1038/s42003-025-09010-x PMC12546842 41125861

[74] Milner DS, Till R, Cadby I, Lovering AL, Basford SM, Saxon EB, Liddell S, Williams LE, Sockett RE. 2014. Ras GTPase-like protein MglA, a controller of bacterial social-motility in Myxobacteria, has evolved to control bacterial predation by *Bdellovibrio*. PLoS Genet 10:e1004253. doi:10.1371/journal.pgen.1004253 PMC3983030 24721965

[75] Kroos L, Wall D, Islam ST, Whitworth DE, Muñoz-Dorado J, Higgs PI, Singer M, Mauriello EMF, Treuner-Lange A, Søgaard-Andersen L, et al.. 2025. Milestones in the development of *Myxococcus xanthus* as a model multicellular bacterium. J Bacteriol 207:e0007125. doi:10.1128/jb.00071-25 PMC12288465 40525847

[76] Liu X, Avramova M, Deme JC, Jones RL, Lundgren CAK, Lea SM, Berks BC. 2025. A shared mechanism for Bacteroidota protein transport and gliding motility. Nat Commun 16:10217. doi:10.1038/s41467-025-65003-8 PMC12635249 41266322

[77] Sun M, Wartel M, Cascales E, Shaevitz JW, Mignot T. 2011. Motor-driven intracellular transport powers bacterial gliding motility. Proc Natl Acad Sci USA 108:7559–7564. doi:10.1073/pnas.1101101108 PMC3088616 21482768

[78] Kaplan M, Chang Y-W, Oikonomou CM, Nicolas WJ, Jewett AI, Kreida S, Dutka P, Rettberg LA, Maggi S, Jensen GJ. 2023. *Bdellovibrio* predation cycle characterized at nanometre-scale resolution with cryo-electron tomography. Nat Microbiol 8:1267–1279. doi:10.1038/s41564-023-01401-2 PMC11061892 37349588

[79] Markelova NY. 2010. Predacious bacteria, *Bdellovibrio* with potential for biocontrol. Int J Hyg Environ Health 213:428–431. doi:10.1016/j.ijheh.2010.08.004 20850380

[80] Jurkevitch E, Minz D, Ramati B, Barel G. 2000. Prey range characterization, ribotyping, and diversity of soil and rhizosphere *Bdellovibrio* spp. isolated on phytopathogenic bacteria. Appl Environ Microbiol 66:2365–2371. doi:10.1128/AEM.66.6.2365-2371.2000 PMC110534 10831412

[81] Saralegui C, Herencias C, Halperin AV, de Dios-Caballero J, Pérez-Viso B, Salgado S, Lanza VF, Cantón R, Baquero F, Prieto MA, et al.. 2022. Strain-specific predation of *Bdellovibrio bacteriovorus* on *Pseudomonas aeruginosa* with a higher range for cystic fibrosis than for bacteremia isolates. Sci Rep 12:10523. doi:10.1038/s41598-022-14378-5 PMC9217795 35732651

[82] Monnappa AK, Dwidar M, Seo JK, Hur JH, Mitchell RJ. 2014. *Bdellovibrio* bacteriovorus inhibits *Staphylococcus aureus* biofilm formation and invasion into human epithelial cells. Sci Rep 4:3811. doi:10.1038/srep03811 PMC3898049 24448451

[83] Iebba V, Totino V, Santangelo F, Gagliardi A, Ciotoli L, Virga A, Ambrosi C, Pompili M, De Biase RV, Selan L, et al.. 2014. *Bdellovibrio bacteriovorus* directly attacks *Pseudomonas aeruginosa* and *Staphylococcus aureus* cystic fibrosis isolates. Front Microbiol 5:280. doi:10.3389/fmicb.2014.00280 PMC4046265 24926292

[84] Koval SF, Bayer ME. 1997. Bacterial capsules: no barrier against *Bdellovibrio*. Microbiology (Reading) 143:749–753. doi:10.1099/00221287-143-3-749 9084160

[85] Koval SF, Hynes SH. 1991. Effect of paracrystalline protein surface layers on predation by *Bdellovibrio bacteriovorus*. J Bacteriol 173:2244–2249. doi:10.1128/jb.173.7.2244-2249.1991 PMC207774 2007549

[86] Ledvina HE, Sayegh R, Carale RO, Burroughs AM, Macklin AR, Azadeh AL, Borja Najera LD, Aravind L, Whiteley AT. 2025. Functional amyloid proteins confer defence against predatory bacteria. Nature 644:197–204. doi:10.1038/s41586-025-09204-7 PMC12419547 40604283

[87] Vidakovic L, Singh PK, Hartmann R, Nadell CD, Drescher K. 2018. Dynamic biofilm architecture confers individual and collective mechanisms of viral protection. Nat Microbiol 3:26–31. doi:10.1038/s41564-017-0050-1 PMC5739289 29085075

[88] Mun W, Upatissa S, Lim S, Dwidar M, Mitchell RJ. 2022. Outer membrane porin F in *E. coli* is critical for effective predation by *bdellovibrio*. Microbiol Spectr 10:e0309422. doi:10.1128/spectrum.03094-22 PMC9769668 36445149

[89] Sayegh R, Ledvina HE, Whiteley AT. 2025. Curli-independent defense against *Bdellovibrio bacteriovorus* in *E. coli*. Microbiol Spectr 13:e0034225. doi:10.1128/spectrum.00342-25 PMC12584612 41020623

[90] Caulton SG, Lambert C, Tyson J, Radford P, Al-Bayati A, Greenwood S, Banks EJ, Clark C, Till R, Pires E, et al.. 2024. *Bdellovibrio bacteriovorus* uses chimeric fibre proteins to recognize and invade a broad range of bacterial hosts. Nat Microbiol 9:214–227. doi:10.1038/s41564-023-01552-2 PMC10769870 38177296

[91] Borkovich KA, Kaplan N, Hess JF, Simon MI. 1989. Transmembrane signal transduction in bacterial chemotaxis involves ligand-dependent activation of phosphate group transfer. Proc Natl Acad Sci USA 86:1208–1212. doi:10.1073/pnas.86.4.1208 PMC286655 2645576

[92] Scherff RH, DeVay JE, Carroll TW. 1965. Ultrastructure of host-parasite relationships involving reproduction of *Bdellovibrio bacteriovorus* in host bacteria. Phytophathology 56:627–632.

[93] Burger A, Drews G, Ladwig R. 1968. The host range and the infectious cycle of a new isolated, on Gram-positive and Gram-negative bacteria parasitising *Bdellovibrio bacteriovorus* strain. Arch Mikrobiol 61:261–279. doi:10.1007/BF00446612 4974080

[94] Fenton AK, Kanna M, Woods RD, Aizawa SI, Sockett RE. 2010. Shadowing the actions of a predator: backlit fluorescent microscopy reveals synchronous nonbinary septation of predatory *Bdellovibrio* inside prey and exit through discrete bdelloplast pores. J Bacteriol 192:6329–6335. doi:10.1128/JB.00914-10 PMC3008530 20935099

[95] Lambert C, Lerner TR, Bui NK, Somers H, Aizawa S-I, Liddell S, Clark A, Vollmer W, Lovering AL, Sockett RE. 2016. Interrupting peptidoglycan deacetylation during *Bdellovibrio* predator-prey interaction prevents ultimate destruction of prey wall, liberating bacterial-ghosts. Sci Rep 6:26010. doi:10.1038/srep26010 PMC4876506 27211869

[96] Harding CJ, Huwiler SG, Somers H, Lambert C, Ray LJ, Till R, Taylor G, Moynihan PJ, Sockett RE, Lovering AL. 2020. A lysozyme with altered substrate specificity facilitates prey cell exit by the periplasmic predator *Bdellovibrio bacteriovorus*. Nat Commun 11:4817. doi:10.1038/s41467-020-18139-8 PMC7511926 32968056

[97] Abram D, Castro e Melo J, Chou D. 1974. Penetration of *Bdellovibrio bacteriovorus* into host cells. J Bacteriol 118:663–680. doi:10.1128/jb.118.2.663-680.1974 PMC246802 4208138

[98] Tahara YO, Mignot T, Miyata M. 2025. Visualization of peptidoglycan layer isolated from gliding diderm bacteria, *Flavobacterium johnsoniae* and *Myxococcus xanthus*, by quick-freeze deep-etch replica electron microscopy. Biophys Physicobiol 22:e220019. doi:10.2142/biophysico.bppb-v22.0019 PMC12536298 41122424

[99] Kuru E, Lambert C, Rittichier J, Till R, Ducret A, Derouaux A, Gray J, Biboy J, Vollmer W, VanNieuwenhze M, et al.. 2017. Fluorescent d-amino-acids reveal bi-cellular cell wall modifications important for *Bdellovibrio bacteriovorus* predation. Nat Microbiol 2:1648–1657. doi:10.1038/s41564-017-0029-y PMC5705579 28974693

[100] Tyson J, Radford P, Lambert C, Till R, Huwiler SG, Lovering AL, Elizabeth Sockett R. 2024. Prey killing without invasion by *Bdellovibrio bacteriovorus* defective for a MIDAS-family adhesin. Nat Commun 15:3078. doi:10.1038/s41467-024-47412-3 PMC11003981 38594280

[101] Lambert C, Ivanov P, Sockett RE. 2010. A transcriptional “scream” early response of *E. coli* prey to predatory invasion by *Bdellovibrio*. Curr Microbiol 60:419–427. doi:10.1007/s00284-009-9559-8 PMC2859166 20024656

[102] Starr MP, Seidler RJ. 1971. The bdellovibrios. Annu Rev Microbiol 25:649–678. doi:10.1146/annurev.mi.25.100171.003245 4949041

[103] Eksztejn M, Varon M. 1977. Elongation and cell division in *Bdellovibrio bacteriovorus*. Arch Microbiol 114:175–181. doi:10.1007/BF00410781 410384

[104] Kessel M, Shilo M. 1976. Relationship of *Bdellovibrio* elongation and fission to host cell size. J Bacteriol 128:477–480. doi:10.1128/jb.128.1.477-480.1976 PMC232876 789349

[105] Ruby EG. 1989. Cell-envelope modifications accompanying intracellular growth of *Bdellovibrio bacteriovorus*, p 17–34. *In* Moulder JW (ed), Intracellular parasitism. CRC Press, Boca Raton.

[106] Odelson DA, Patterson MA, Hespell RB. 1982. Periplasmic enzymes in *Bdellovibrio bacteriovorus* and *Bdellovibrio stolpii*. J Bacteriol 151:756–763. doi:10.1128/jb.151.2.756-763.1982 PMC220322 6124531

[107] Fackrell HB, Robinson J. 1973. Purification and characterization of a lytic peptidase produced by *Bdellovibrio bacteriovorus* 6-5-S. Can J Microbiol 19:659–666. doi:10.1139/m73-107 4709167

[108] Tudor JJ, McCann MP, Acrich IA. 1990. A new model for the penetration of prey cells by bdellovibrios. J Bacteriol 172:2421–2426. doi:10.1128/jb.172.5.2421-2426.1990 PMC208878 2185219

[109] Ruby EG, Rittenberg SC. 1983. Differentiation after premature release of intraperiplasmically growing *Bdellovibrio bacteriovorous*. J Bacteriol 154:32–40. doi:10.1128/jb.154.1.32-40.1983 PMC217427 6403505

[110] Santin YG, Sogues A, Bourigault Y, Remaut HK, Laloux G. 2024. Lifecycle of a predatory bacterium vampirizing its prey through the cell envelope and S-layer. Nat Commun 15:3590. doi:10.1038/s41467-024-48042-5 PMC11055950 38678033

[111] Hocher A, Laursen SP, Radford P, Tyson J, Lambert C, Stevens KM, Montoya A, Shliaha PV, Picardeau M, Sockett RE, et al.. 2023. Histones with an unconventional DNA-binding mode in vitro are major chromatin constituents in the bacterium *Bdellovibrio bacteriovorus*. Nat Microbiol 8:2006–2019. doi:10.1038/s41564-023-01492-x PMC10627809 37814071

[112] Borgnia MJ, Subramaniam S, Milne JLS. 2008. Three-dimensional imaging of the highly bent architecture of *Bdellovibrio bacteriovorus* by using cryo-electron tomography. J Bacteriol 190:2588–2596. doi:10.1128/JB.01538-07 PMC2293184 18203829

[113] Butan C, Hartnell LM, Fenton AK, Bliss D, Sockett RE, Subramaniam S, Milne JLS. 2011. Spiral architecture of the nucleoid in *Bdellovibrio bacteriovorus*. J Bacteriol 193:1341–1350. doi:10.1128/JB.01061-10 PMC3067633 21148724

[114] Hu Y, Schwab S, Deiss S, Escudeiro P, van Heesch T, Joiner JD, Vreede J, Hartmann MD, Lupas AN, Alvarez BH, et al.. 2024. Bacterial histone HBb from *Bdellovibrio bacteriovorus* compacts DNA by bending. Nucleic Acids Res 52:8193–8204. doi:10.1093/nar/gkae485 PMC11317129 38864377

[115] Kaljević J, Saaki TNV, Govers SK, Remy O, van Raaphorst R, Lamot T, Laloux G. 2021. Chromosome choreography during the non-binary cell cycle of a predatory bacterium. Curr Biol 31:3707–3720. doi:10.1016/j.cub.2021.06.024 PMC8445325 34256020

[116] Pląskowska K, Zakrzewska-Czerwińska J. 2023. Chromosome structure and DNA replication dynamics during the life cycle of the predatory bacterium *Bdellovibrio bacteriovorus*. FEMS Microbiol Rev 47:fuad057. doi:10.1093/femsre/fuad057 PMC11318664 37791401

[117] Laloux G. 2019. Shedding light on the cell biology of the predatory bacterium *Bdellovibrio bacteriovorus*. Front Microbiol 10:3136. doi:10.3389/fmicb.2019.03136 PMC6985089 32038570

[118] Makowski Ł, Trojanowski D, Till R, Lambert C, Lowry R, Sockett RE, Zakrzewska-Czerwińska J. 2019. Dynamics of chromosome replication and its relationship to predatory attack lifestyles in *Bdellovibrio bacteriovorus*. Appl Environ Microbiol 85:e00730-19. doi:10.1128/AEM.00730-19 PMC6606864 31076424

[119] Makowski Ł, Trojanowski D, Zakrzewska-Czerwińska J. 2020. Live-cell imaging of the life cycle of bacterial predator *Bdellovibrio bacteriovorus* using time-lapse fluorescence microscopy. J Vis Exp 2020:159. doi:10.3791/61105 32449729

[120] Lowry RC, Milner DS, Al-Bayati AMS, Lambert C, Francis VI, Porter SL, Sockett RE. 2019. Evolutionary diversification of the RomR protein of the invasive deltaproteobacterium, *Bdellovibrio bacteriovorus*. Sci Rep 9:5007. doi:10.1038/s41598-019-41263-5 PMC6428892 30899045

[121] Milner DS, Ray LJ, Saxon EB, Lambert C, Till R, Fenton AK, Sockett RE. 2020. DivIVA controls progeny morphology and diverse ParA proteins regulate cell division or gliding motility in *Bdellovibrio bacteriovorus*. Front Microbiol 11:542. doi:10.3389/fmicb.2020.00542 PMC7186360 32373080

[122] Kaljević J, Tesseur C, Le TBK, Laloux G. 2023. Cell cycle-dependent organization of a bacterial centromere through multi-layered regulation of the ParABS system. PLoS Genet 19:e1010951. doi:10.1371/journal.pgen.1010951 PMC10547168 37733798

[123] Remy O, Santin YG, Jonckheere V, Tesseur C, Kaljević J, Van Damme P, Laloux G. 2024. Distinct dynamics and proximity networks of hub proteins at the prey-invading cell pole in a predatory bacterium. J Bacteriol 206:e0001424. doi:10.1128/jb.00014-24 PMC11025332 38470120

[124] Tudor JJ, Davis JJ, Panichella M, Zwolak A. 2008. Isolation of predation-deficient mutants of *Bdellovibrio bacteriovorus* by using transposon mutagenesis. Appl Environ Microbiol 74:5436–5443. doi:10.1128/AEM.00256-08 PMC2546638 18621871

[125] Medina AA, Shanks RM, Kadouri DE. 2008. Development of a novel system for isolating genes involved in predator-prey interactions using host independent derivatives of *Bdellovibrio bacteriovorus* 109J. BMC Microbiol 8:33. doi:10.1186/1471-2180-8-33 PMC2277423 18284687

[126] Duncan MC, Gillette RK, Maglasang MA, Corn EA, Tai AK, Lazinski DW, Shanks RMQ, Kadouri DE, Camilli A. 2019. High-throughput analysis of gene function in the bacterial predator *Bdellovibrio bacteriovorus*. mBio 10:e01040-19. doi:10.1128/mBio.01040-19 PMC6561027 31186325

[127] Cotter TW, Thomashow MF. 1992. A conjugation procedure for *Bdellovibrio bacteriovorus* and its use to identify DNA sequences that enhance the plaque-forming ability of a spontaneous host-independent mutant. J Bacteriol 174:6011–6017. doi:10.1128/jb.174.19.6011-6017.1992 PMC207665 1400153

[128] Steyert SR, Pineiro SA. 2007. Development of a novel genetic system to create markerless deletion mutants of *Bdellovibrio bacteriovorus*. Appl Environ Microbiol 73:4717–4724. doi:10.1128/AEM.00640-07 PMC1951038 17557848

[129] Fenton AK, Lambert C, Wagstaff PC, Sockett RE. 2010. Manipulating each MreB of *Bdellovibrio bacteriovorus* gives diverse morphological and predatory phenotypes. J Bacteriol 192:1299–1311. doi:10.1128/JB.01157-09 PMC2820843 20023029

[130] Chang C-Y, Hobley L, Till R, Capeness M, Kanna M, Burtt W, Jagtap P, Aizawa S-I, Sockett RE. 2011. The *Bdellovibrio bacteriovorus* twin-arginine transport system has roles in predatory and prey-independent growth. Microbiology 157:3079–3093. doi:10.1099/mic.0.052449-0 21903758

[131] Roschanski N, Strauch E. 2011. Assessment of the mobilizable vector plasmids pSUP202 and pSUP404.2 as genetic tools for the predatory bacterium *Bdellovibrio bacteriovorus*. Curr Microbiol 62:589–596. doi:10.1007/s00284-010-9748-5 PMC3021704 20824276

[132] Mukherjee S, Brothers KM, Shanks RMQ, Kadouri DE. 2016. Visualizing *Bdellovibrio bacteriovorus* by using the tdTomato fluorescent protein. Appl Environ Microbiol 82:1653–1661. doi:10.1128/AEM.03611-15 PMC4784026 26712556

[133] Cotter TW, Thomashow MF. 1992. Identification of a *Bdellovibrio bacteriovorus* genetic locus, hit, associated with the host-independent phenotype. J Bacteriol 174:6018–6024. doi:10.1128/jb.174.19.6018-6024.1992 PMC207666 1400154

[134] Lambert C, Cadby IT, Till R, Bui NK, Lerner TR, Hughes WS, Lee DJ, Alderwick LJ, Vollmer W, Sockett RE, Lovering AL. 2015. Ankyrin-mediated self-protection during cell invasion by the bacterial predator *Bdellovibrio bacteriovorus*. Nat Commun 6:8884. doi:10.1038/ncomms9884 PMC4686830 26626559

[135] Capeness MJ, Lambert C, Lovering AL, Till R, Uchida K, Chaudhuri R, Alderwick LJ, Lee DJ, Swarbreck D, Liddell S, Aizawa S-I, Sockett RE. 2013. Activity of *Bdellovibrio Hit* locus proteins, Bd0108 and Bd0109, links type IVa pilus extrusion/retraction status to prey-independent growth signalling. PLoS One 8:e79759. doi:10.1371/journal.pone.0079759 PMC3818213 24224002

[136] Roschanski N, Klages S, Reinhardt R, Linscheid M, Strauch E. 2011. Identification of genes essential for prey-independent growth of *Bdellovibrio bacteriovorus* HD100. J Bacteriol 193:1745–1756. doi:10.1128/JB.01343-10 PMC3067643 21278289

[137] Barel G, Jurkevitch E. 2001. Analysis of phenotypic diversity among host-independent mutants of *Bdellovibrio bacteriovorus* 109J. Arch Microbiol 176:211–216. doi:10.1007/s002030100312 11511869

[138] Medina AA, Kadouri DE. 2009. Biofilm formation of *Bdellovibrio bacteriovorus* host-independent derivatives. Res Microbiol 160:224–231. doi:10.1016/j.resmic.2009.02.001 19223013

[139] Hobley L, Lerner TR, Williams LE, Lambert C, Till R, Milner DS, Basford SM, Capeness MJ, Fenton AK, Atterbury RJ, et al.. 2012. Genome analysis of a simultaneously predatory and prey-independent, novel *Bdellovibrio bacteriovorus* from the River Tiber, supports in silico predictions of both ancient and recent lateral gene transfer from diverse bacteria. BMC Genomics 13:670. doi:10.1186/1471-2164-13-670 PMC3539863 23181807

[140] Galperin MY. 2005. A census of membrane-bound and intracellular signal transduction proteins in bacteria: bacterial IQ, extroverts and introverts. BMC Microbiol 5:35. doi:10.1186/1471-2180-5-35 PMC1183210 15955239

[141] Hobley L, Fung RKY, Lambert C, Harris M, Dabhi JM, King SS, Basford SM, Uchida K, Till R, Ahmad R, et al.. 2012. Discrete cyclic di-GMP-dependent control of bacterial predation versus axenic growth in *Bdellovibrio bacteriovorus*. PLoS Pathog 8:e1002493. doi:10.1371/journal.ppat.1002493 PMC3271064 22319440

[142] Lowry RC, Hallberg ZF, Till R, Simons TJ, Nottingham R, Want F, Sockett RE, Hammond MC, Lambert C. 2022. Production of 3’,3’-cGAMP by a *Bdellovibrio bacteriovorus* promiscuous GGDEF enzyme, Bd0367, regulates exit from prey by gliding motility. PLoS Genet 18:e1010164. doi:10.1371/journal.pgen.1010164 PMC9140294 35622882

[143] Rotem O, Nesper J, Borovok I, Gorovits R, Kolot M, Pasternak Z, Shin I, Glatter T, Pietrokovski S, Jenal U, et al.. 2016. An extended cyclic Di-GMP network in the predatory bacterium *Bdellovibrio bacteriovorus*. J Bacteriol 198:127–137. doi:10.1128/JB.00422-15 PMC4686195 26324450

[144] Sathyamoorthy R, Kushmaro Y, Rotem O, Matan O, Kadouri DE, Huppert A, Jurkevitch E. 2021. To hunt or to rest: prey depletion induces a novel starvation survival strategy in bacterial predators. ISME J 15:109–123. doi:10.1038/s41396-020-00764-2 PMC7852544 32884113

[145] Meek RW, Cadby IT, Moynihan PJ, Lovering AL. 2019. Structural basis for activation of a diguanylate cyclase required for bacterial predation in *Bdellovibrio*. Nat Commun 10:4086. doi:10.1038/s41467-019-12051-6 PMC6733907 31501441

[146] Barel G, Sirota A, Volpin H, Jurkevitch E. 2005. Fate of predator and prey proteins during growth of *Bdellovibrio bacteriovorus* on *Escherichia coli* and *Pseudomonas syringae* prey. J Bacteriol 187:329–335. doi:10.1128/JB.187.1.329-335.2005 PMC538828 15601717

[147] Karunker I, Rotem O, Dori-Bachash M, Jurkevitch E, Sorek R. 2013. A global transcriptional switch between the attack and growth forms of *Bdellovibrio bacteriovorus*. PLoS One 8:e61850. doi:10.1371/journal.pone.0061850 PMC3627812 23613952

[148] Dori-Bachash M, Dassa B, Pietrokovski S, Jurkevitch E. 2008. Proteome-based comparative analyses of growth stages reveal new cell cycle-dependent functions in the predatory bacterium *Bdellovibrio bacteriovorus*. Appl Environ Microbiol 74:7152–7162. doi:10.1128/AEM.01736-08 PMC2592910 18836011

[149] Mihajlovic L, Hofacker LM, Lindner F, Jayakumar P, Diepold A, Huwiler SG. 2025. A molecular toolbox to modulate gene expression and protein secretion in the bacterial predator *Bdellovibrio bacteriovorus*. PLoS Genet 21:e1011935. doi:10.1371/journal.pgen.1011935 PMC12622784 41212898

[150] de Pierpont C, Derneden B, Remy O, Laloux G. 2025. Expanding the genetic toolbox of the obligate predatory bacterium *Bdellovibrio bacteriovorus* with inducible gene expression and CRISPR interference. Microlife 6:uqaf021. doi:10.1093/femsml/uqaf021 PMC12448681 40977858

[151] Lerner TR, Lovering AL, Bui NK, Uchida K, Aizawa S, Vollmer W, Sockett RE. 2012. Specialized peptidoglycan hydrolases sculpt the intra-bacterial niche of predatory *Bdellovibrio* and increase population fitness. PLoS Pathog 8:e1002524. doi:10.1371/journal.ppat.1002524 PMC3276566 22346754

[152] Banks EJ, Lambert C, Mason SS, Tyson J, Radford PM, McLaughlin C, Lovering AL, Sockett RE. 2023. An MltA-like lytic transglycosylase secreted by *Bdellovibrio bacteriovorus* cleaves the prey septum during predatory invasion. J Bacteriol 205:e0047522. doi:10.1128/jb.00475-22 PMC10127604 37010281

[153] Nelson DR, Rittenberg SC. 1981. Partial characterization of lipid A of intraperiplasmically grown *Bdellovibrio bacteriovorus*. J Bacteriol 147:869–874. doi:10.1128/jb.147.3.869-874.1981 PMC216123 7024250

[154] Nelson DR, Rittenberg SC. 1981. Incorporation of substrate cell lipid A components into the lipopolysaccharide of intraperiplasmically grown *Bdellovibrio bacteriovorus*. J Bacteriol 147:860–868. doi:10.1128/jb.147.3.860-868.1981 PMC216122 7024249

[155] Schwudke D, Linscheid M, Strauch E, Appel B, Zahringer U, Moll H, Muller M, Brecker L, Gronow S, Lindner B. 2003. The obligate predatory *Bdellovibrio bacteriovorus* possesses a neutral lipid A containing α-D-mannoses that replace phosphate residues. Similarities and differences between the lipid as and the lipopolysaccharides of the wild type strain *B. bacteriovorus* HD100 and its host-independent derivative HI100. J Biol Chem 278:27502–27512. doi:10.1074/jbc.M303012200 12743115

[156] Parr RJ, Santin YG, Ratkevičiūte G, Caulton SG, Radford P, Gurvič D, Jenkins M, Doyle MT, Mead L, Silale A, et al.. 2025. A porin-like protein used by bacterial predators defines a wider lipid-trapping superfamily. Nat Commun 16:6213. doi:10.1038/s41467-025-61633-0 PMC12228718 40617869

